# Supplementary material for: Association between oral microbiome and seven types of cancers in East Asian population: a two-sample Mendelian randomization analysis
Source: Front Mol Biosci. 2023 Nov 21;10:1327893. doi: 10.3389/fmolb.2023.1327893 (PMC10702768; doi:10.3389/fmolb.2023.1327893)
Supplement: Supplementary file 1 [file DataSheet1.ZIP › Supplementary Materials/MR plots of saliva/saliva═╝/Prostate cancer/pheno.2207_to_prostate cancer_forest.pdf]

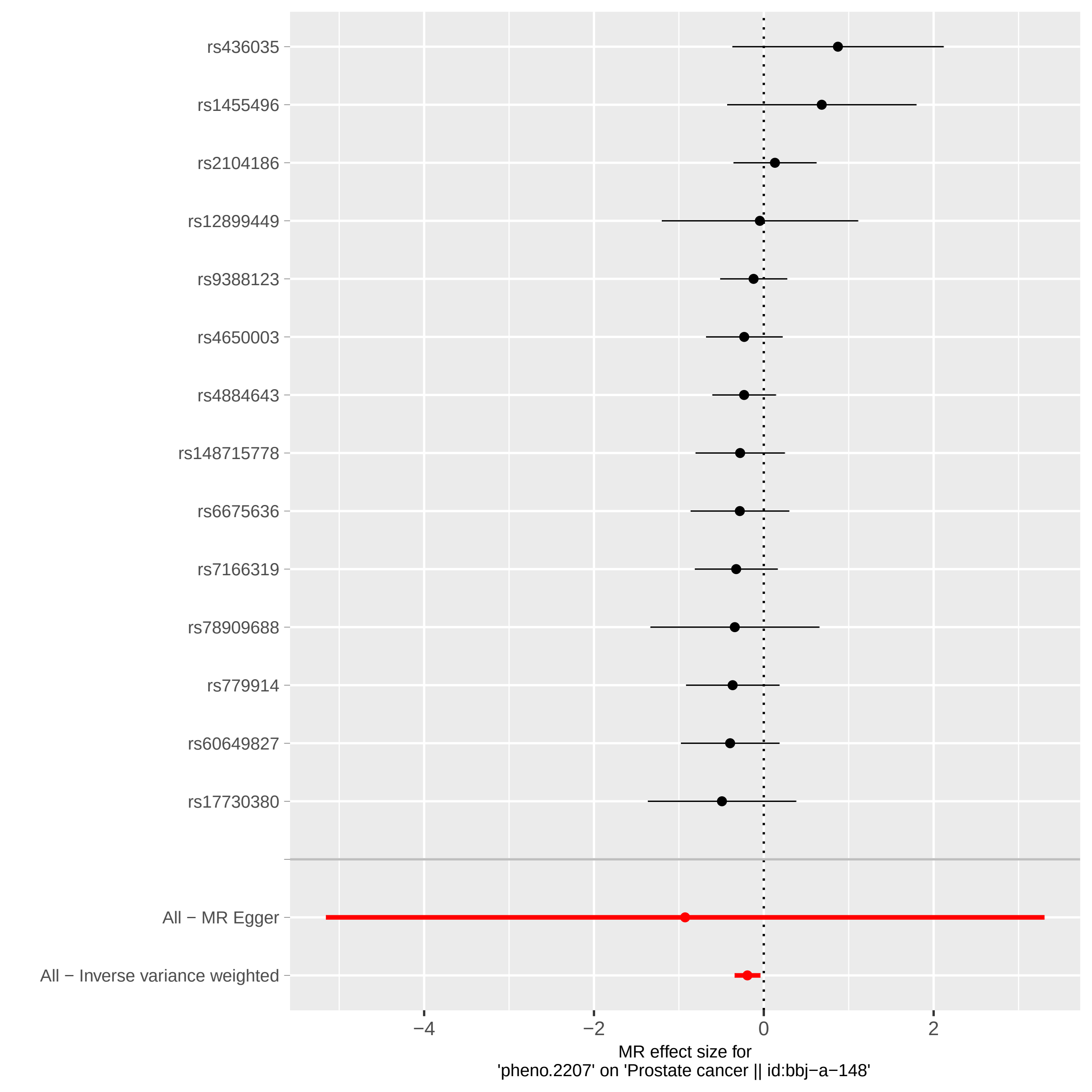
rs436035
rs1455496
rs2104186
rs12899449
rs9388123
rs4650003
rs4884643
rs148715778
rs6675636
rs7166319
rs78909688
rs779914
rs60649827
rs17730380
All − MR Egger
All − Inverse variance weighted
−4
−2
0
2
MR effect size for
'pheno.2207' on 'Prostate cancer || id:bbj−a−148'
